# Introducing Catastrophe-QSAR. Application on Modeling Molecular Mechanisms of Pyridinone Derivative-Type HIV Non-Nucleoside Reverse Transcriptase Inhibitors

**DOI:** 10.3390/ijms12129533

**Published:** 2011-12-20

**Authors:** Mihai V. Putz, Marius Lazea, Ana-Maria Putz, Corina Duda-Seiman

**Affiliations:** 1 Laboratory of Computational and Structural Physical Chemistry, Biology & Chemistry Department, West University of Timisoara, Str. Pestalozzi No. 16, 300115 Timisoara, Romania; E-Mails: laz_marius2@yahoo.com (M.L.), cori_mam@yahoo.com (C.D.-S.); 2 Institute of Chemistry Timisoara of the Romanian Academy, 24 Mihai Viteazul Bld, RO-300223, Timisoara, Romania

**Keywords:** Thom’s catastrophe polynomials, statistical factors, minimum statistical paths, QSAR structural domains, HIV-1 inhibitory activity

## Abstract

The classical method of quantitative structure-activity relationships (QSAR) is enriched using non-linear models, as Thom’s polynomials allow either uni- or bi-variate structural parameters. In this context, catastrophe QSAR algorithms are applied to the anti-HIV-1 activity of pyridinone derivatives. This requires calculation of the so-called relative statistical power and of its minimum principle in various QSAR models. A new index, known as a statistical relative power, is constructed as an Euclidian measure for the combined ratio of the Pearson correlation to algebraic correlation, with normalized t-Student and the Fisher tests. First and second order inter-model paths are considered for mono-variate catastrophes, whereas for bi-variate catastrophes the direct minimum path is provided, allowing the QSAR models to be tested for predictive purposes. At this stage, the max-to-min hierarchies of the tested models allow the interaction mechanism to be identified using structural parameter succession and the typical catastrophes involved. Minimized differences between these catastrophe models in the common structurally influential domains that span both the trial and tested compounds identify the “optimal molecular structural domains” and the molecules with the best output with respect to the modeled activity, which in this case is human immunodeficiency virus type 1 HIV-1 inhibition. The best molecules are characterized by hydrophobic interactions with the HIV-1 p66 subunit protein, and they concur with those identified in other 3D-QSAR analyses. Moreover, the importance of aromatic ring stacking interactions for increasing the binding affinity of the inhibitor-reverse transcriptase ligand-substrate complex is highlighted.

## 1. Introduction

Among the mathematical theories that model open-system dynamics, Thom’s theory of catastrophes has acquired much popularity for its simple yet valuable description of the system-environment interaction that includes phenomena such as steady state equilibrium and life cycles [1]. In particular, biological systems come first under catastrophe modeling because they display a causal action-reaction response to various natural or imposed constraining limits. As an example, the reactions of organisms to vital toxicological threats were developed into the survival attractor concept by employing butterfly bifurcation phenomenology, which is closely related to the cusp catastrophe, thus revealing the close connection with the turning points around singularity points of the fundamental central field laws of attraction [2]. The cusp catastrophe was further implemented in the physiological processes of predation and generation, thus giving mathematical support to Heidegger’s philosophical concept of *entity* and having the major consequence of translating the ontological entities into computer language [3]. Following this line of application, Jungian psychology entered the topological approach phase through modeling personal unconscious and conscious states using the swallowtail catastrophe [4]. As a consequence, neuro-self-organization was advanced by reduction to cusp synergetics as an archetypal precursor of epileptic seizures [5]. Nevertheless, in chemistry the catastrophe approach enters through the need to unitarily characterize elementary processes such as chemical bonding, leading to the so-called bonding evolution theory and reformulation of the electronic localization functions [6,7]. In the last decade, catastrophe theory was successfully grounded on Hilbert space modeling with the density matrix and non-linear evolution as specific tools for the non-commutative (quantum) systems [8]. At this point, the interesting connection with the linear superposition of quantum states may be generalized in a non-linear manner with direct correspondence for widespread quantitative structure-activity relationship (QSAR) treatments of the “birth and death of an organism”.

In this context, the present contribution provides *in silico* assistance to clinical efforts in current antiretroviral therapy by contributing to the development of a given class of actual anti-HIV-1 compounds and identifying their viral inhibitory mechanisms and influential structural factors. Continuous efforts both in theory and in clinical practice are made to provide new and valid data for HIV infection management. Note that acquired immunodeficiency deficiency syndrome (AIDS) was first recognized in 1981. Only 25 compounds have been approved for use in HIV infected patients, and they are distributed among several classes of antiretroviral drug types [9,10]: nucleoside reverse transcriptase inhibitors (NRTIs); nucleotide reverse transcriptase inhibitors (NtRTIs); non-nucleoside reverse transcriptase inhibitors (NNRTIs); protease inhibitors (PIs); cell entry (or fusion) inhibitors (FIs); co-receptor inhibitors (CRIs); and integrase inhibitors (INIs). Among these, it is well known that most NNRTIs have a low genetic barrier to resistance, *i.e.*, high viral resistance may be induced by a single mutation at the NNRTI binding site [11]. It is this particular feature that makes NNRTIs so well adapted for a comprehensive catastrophe theory application. Although NNRTIs are an open battlefield for research, being highly active in naïve and drug-resistant HIV infected patients [12], QSAR methods are cost-effective approaches to developing new and potent molecules with increased anti-HIV activity [13–23]. As a viable alternative to the available 3D-QSARs, the present endeavor makes the first steps toward generalizing multi-linear QSAR to non-linear catastrophe QSAR analysis and toward providing a conceptual-computational framework in which both the interactions occurring between the pyridinone derivatives and the NNRTI binding site and the structural domains influential for HIV-1 RT inhibitory activity are accounted for [24].

## 2. Background Theories

### 2.1. QSAR Phenomenology

The fundamental problem of structure-activity analysis may be described as follows: given a congener set of *N*-compounds/molecules with measured/observed activity (*A*) one searches for the best correlation of it with the structural (intrinsic, internal) molecular information quantified by *M*-properties (such as hydrophobicity, polarization, total energy), classically presented in multi-linear form [25–31]:

(1) $$Y=b_{0}+b_{1}X_{1}+\ldots +b_{k}X_{k}+\ldots +b_{M}X_{M}$$

Equation (1) has some basic features, namely:

- *Y* stands for the computed activity, not the observed activity, from the statistical characteristics of the present approach; thus the validation of Equation (1) should be done for another (preferably external or testing) set of compounds with which the predictive power of Equation (1) is tested.
- Because the right side of Equation (1) unfolds as a linear summation of the structural characteristics, it corresponds in fact with the quantum superposition principle, which provides a global Eigen-solution for a quantum system from its particular realization in orthogonal or projective sub-space; from where the need arises for structural indices *X*_1_, ..., *X_M_* to be either linearly independent or orthogonal in algebraic space built from their associate vectors presented in Table 1.

However, in order for the chemical structure be correlated with bio-, eco-, or pharmacological activity in an analytical manner (from where the name Quantitative Structure-Activity Relationship arises) that has sense for the ligand-receptor interaction under study, the Organization for Economic Cooperation and Development (OECD) developed the so-called QSAR-OECD principles, which have already been adopted by the EU Parliament as the official guidelines for further regulation of compounds in the European Union. They are, in short [32]:

- QSAR 1: a defined endpoint
- QSAR-2: an unambiguous algorithm
- QSAR-3: a defined domain of applicability
- QSAR-4: appropriate measures of goodness-of–fit, robustness and predictivity
- QSAR-5: a mechanistic interpretation, if possible

Put differently, they express the essence of the chemical modeling of biological effects while relaying (Husserl-Russell) knowledge phenomenology in a more general manner [33]:

- QSAR-1. why does one do modeling ?
- QSAR-2. how does one do modeling ?
- QSAR-3. with what tools do I model ?
- QSAR-4. how reliable is what I modeled ?
- QSAR-5. what knowledge did the model provide ?

Therefore, although the backbone of QSAR modeling is based on equation (1), one should be aware that it represents, despite the innumerable extant studies, only one type of model—the multi-linear type. It is therefore worth refreshing QSAR studies by exploring other ways of combining the structural parameters that cause the observed biological activity. However, although it is clear that non-linear QSAR is the next generation of correlations, one should not search arbitrarily or randomly while having at hand a well-designed theory of non-linear modeling of natural phenomena: Thom’s catastrophe theory, the basic assumptions and main working tools of which are presented next.

### 2.2. Thom’s Catastrophe Theory

René Thom’s catastrophe theory basically describes how, for a given system, continuous action on the *control space* (*C^k^*), parameterized by *C_k_*’s, provides a sudden change in its *behavior space* (*I^m^*), described by *x_m_* variables through stable singularities of the smooth map [34,35]

(2) $$\eta (c_{k},x_{m}):C^{k}\times I^{m}\to R$$

with *η*(*c_k_*, *x_m_*) called the *generic potential* of the system. Therefore, catastrophes are given by the set of *critical points* (*c_k_*, *x_m_*) for which the field gradient of the generic potential vanishes

(3) $$M^{k\times m}=\left\{(c_{k},x_{m})\in C^{k}\times I^{m}|\nabla_{x_{m}}\eta (c_{k},x_{m})=0\right\}$$

or, more rigorously: a catastrophe is a singularity of the map *M^k^*^×^*^m^* → *C^k^*.

Next, depending on the number of parameters in space *C^k^* (also called the *co-dimension*, *k*) and on the number of variables in space *I^m^* (also called the *co-rank*, *m*), Thom classified the generic potentials (or maps) given by Equation (2) as seven unfolding elementary (in the sense of universal) catastrophes, *i.e.*, providing the multi-variable (with the co-rank up to two) and multi-parametrical (with the co-dimension up to four) polynomials listed in Table 2. Going to the higher derivatives of the generic potential (the fields), the control parameter c_k_* for which the Laplacian of the generic potential vanishes

(4) $$\Delta_{x}\eta (c_{k}*,x_{m})=0$$

gives the *bifurcation point*. Consequently, the set of control parameters *c*^#^ for which the Laplacian of a critical point is non-zero defines the *domain of stability* of the critical point. It is clear now that small perturbations of *η*(c*, *x*) bring the system from one domain of stability to another; otherwise, the system is located within a *domain of structural stability*.

Remarkably, the cases described above correspond to the equilibrium limit of the dynamical (non-equilibrium) evolution of an open system

(5) $$F\left(c_{k};t;\eta (c_{k};x_{m});\frac{\partial \eta (c_{k};x_{m})}{\partial t},\ldots \right)=0$$

where the behavior space is further parameterized by the temporal paths *x_m_*(*c_k_*, *t*). The connection with equilibrium is recovered through the stationary time regime imposed on the critical points. In this way, the set of points giving a critical point in the stationary *t* → +∞ regime (the so-called ω*-limit*) corresponds to *an attractor*, and it forms a *basin*, whereas the stationary regime *t*→ −∞ (the so-called *α-limit*) describes *a repellor*. In this way, the catastrophe polynomials may be regarded either as an asymptotic solution of a dynamical evolutionary system or as a steady state solution allowing the quasi-equilibrium of the ligand-receptor or inhibitor-organism interactions to be described. However, in complex binding systems with multiple evolutionary phases, e.g., the HIV-1 life cycle, the possibility of “linking” the various classes of catastrophes themselves may provide a striking analytical approach to the dynamics and mutational sensitivity of the studied interaction that starts with the actual catastrophe-QSAR method.

## 3. Catastrophe-QSAR Method

Aiming to construct a QSAR rationale from the elementary catastrophes, the next steps are implemented:

- Assuming the vectorial form of activities and of associated QSARs are according to Table 2, Table 3 showing catastrophe-QSAR is thereby formed.
- Determine the norms for each model
  (6) $$\left\||Y\rangle \right\|=\sqrt{\langle Y|Y\rangle }=\sqrt{\sum_{i=1}^{N}y_{i}^{2}}$$
- Calculate the algebraic correlation factor for each model [31]
  (7) $$R_{ALG}=\frac{\left\||Y\rangle \right\|}{\left\||A\rangle \right\|}=\sqrt{\frac{\sum_{i=1}^{N}y_{i}^{2}}{\sum_{i=1}^{N}A_{i}^{2}}}$$
- Calculate the so-called “*statistical relative power*” index for each model with each set of descriptors
  (8) $$\Pi =\sqrt{r^{2}+t^{2}+f^{2}}$$
  where the components are defined as follows:
  - relative index of correlation:
    (9) $$r=\frac{R_{ALG}}{R_{Pearson}}$$
  - relative index for Student’s *t*-test
    (10) $$t=\frac{t_{Computed}}{t_{\begin{array}{l} Tabulated\\ (1-\alpha =0.99;\\ N-M-2) \end{array}}}$$
  - relative index for Fisher’s test
    (11) $$f=\frac{F_{Computed}}{F_{\begin{array}{l} Tabulated\\ (1-\alpha =0.99;\\ M,N-M-1) \end{array}}}$$
- Determine the generalized Euclidian distances between corresponding type-I and type-II models employing different descriptors
  (12) $$\Delta \Pi =\sqrt{{\left(r-r^{\prime }\right)}^{2}+{\left(t-t^{\prime }\right)}^{2}+{\left(f-f^{\prime }\right)}^{2}}$$
  and establish formal matrices for the models’ differences for single descriptors, respectively
  (13) $$\Delta^{2}\Pi_{I(X_{1},X_{2})}=\left|\Delta \Pi_{I(X_{1})}-\Delta \Pi_{I(X_{2})}\right|$$
  where
  (14) $$\begin{array}{l} \Delta \Pi_{I(X=X_{1}\vee X_{2})}\\ =\left(\begin{array}{cccc} {\mathrm{QSAR}}_{I(X)}-F_{\left(X\right)} & {\mathrm{QSAR}}_{I(X)}-C_{\left(x\right)} & {\mathrm{QSAR}}_{I(X)}-{ST}_{\left(X\right)} & {\mathrm{QSAR}}_{I(X)}-B_{\left(X\right)}\\ & F_{\left(X\right)}-C_{\left(X\right)} & F_{\left(X\right)}-{ST}_{\left(X\right)} & F_{\left(X\right)}-B_{\left(X\right)}\\ & & C_{\left(X\right)}-{ST}_{\left(X\right)} & C_{\left(X\right)}-B_{\left(X\right)}\\ & & & {ST}_{\left(X\right)}-B_{\left(X\right)} \end{array}\right) \end{array}$$
  and for pair descriptors
  (15) $$\begin{array}{l} \Delta \Pi_{II(X_{1}\wedge X_{2})}\\ =\left(\begin{array}{ccc} {\mathrm{QSAR}}_{II(X_{1},X_{2})}-{HU}_{\left(X_{1},X_{2}\right)} & {\mathrm{QSAR}}_{II(X_{1},X_{2})}-{EU}_{\left(X_{1},X_{2}\right)} & {\mathrm{QSAR}}_{II(X_{1},X_{2})}-{PU}_{\left(X_{1},X_{2}\right)}\\ & {HU}_{\left(X_{1},X_{2}\right)}-{EU}_{\left(X_{1},X_{2}\right)} & {HU}_{\left(X_{1},X_{2}\right)}-{PU}_{\left(X_{1},X_{2}\right)}\\ & & {EU}_{\left(X_{1},X_{2}\right)}-{PU}_{\left(X_{1},X_{2}\right)} \end{array}\right) \end{array}$$
- Identify all minimum paths across all differences ΔΠ*_I_* _(_*_X_*__1_∨_*_X_*__2_)_, Δ^2^Π*_I_*_(_*_X_*__1_,_*_X_*__2_)_ and ΔΠ*_II_*_(_*_X_*__1_∧_*_X_*__2_)_ for a given set of descriptors (*X*_1_, *X*_2_)
  (16) $$\{\begin{array}{l} \delta \left\{\Delta \Pi_{I(X)}\right\}=0\\ \delta \left\{\Delta^{2}\Pi_{I(X_{1}\vee X_{2})}\right\}=0\\ \delta \left\{\Delta \Pi_{II(X_{1}\wedge X_{2})}\right\}=0 \end{array}$$
  The combination of descriptors that fulfills this system provides the *molecular mechanism* of the interaction. The correlation models involved are ordered according to their relative statistical power within the same molecular mechanism, thereby providing the *best models*. Because pair-descriptors are primarily involved in the present analysis, one can consider the first two such “waves” and their best correlation models up to the second order minimum paths, as in Equation (16).
- For selected correlation models, in either structure-driven or molecular mechanistic “waves,” one employs them to compute the associated predicted activities for test molecules and to provide the statistics regarding the observed activity. If the obtained relative statistical power is close to those characteristic for the trial set of molecules, then these models may be validated for the specific eco-, bio-, or pharmacological problem. Moreover, further insight will be provided by the analysis of the catastrophe shape of the models involved and discussed accordingly.

Nevertheless, more Catastrophe Theory insights and the natural consequence on statistical (Pearson) correlation behavior may be found in Appendix.

## 4. Application to Non-Nucleoside Reverse Transcriptase Pyridinone Inhibitors

### 4.1. Input Data

**Figure 1:**
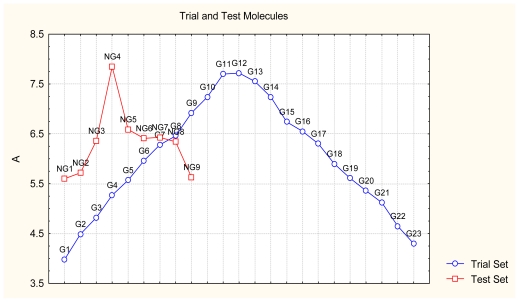
Gaussian (G) and non-Gaussian (NG) screening of the observed activities of the working molecules in Table 4 grouped into trial and test congener series.

As a working molecular series, the interesting series of pyridinone derivatives in Table 4 is herein employed [24] because of their potential for improving and complementing the currently available four NNRTIs that have been approved by the U.S. FDA for HIV/AIDS treatment (Nevirapine-Viramune^®^, Delavirdine-Rescriptor^®^, Efavirenz-Sustiva^®^, Etravirine-Intelence^®^), all of which bind to the hydrophobic pocket of HIV-1 reverse transcriptase [38]. The pyridinone derivatives were divided into a training set of 23 compounds and a test set of 9 compounds according to the methods of normal/Gaussian (G) and non-normal/non-Gaussian (NG) fitted activity [39–41] (Figure 1).

### 4.2. Results and Discussion

The catastrophe-QSAR algorithm of Section 3 was applied to the molecules of Table 4, and the trial results are presented in Tables 5–9.

For the trial set of molecules from Figure 1 and Table 4, the results in Tables 5 and 6 can be interpreted as follows:

- First, it is clear that consideration of the catastrophe (polynomial) correlations is an improvement over the old multi-linear QSAR statistics (see also Appendix-A2).
- The hydrophobicity indicator gives generally low correlations with any polynomial (linear, multilinear or catastrophe) approach, being a quite irrelevant linear QSAR descriptor (Table 5) but improving up to twice its influence within the swallow tail and butterfly phenomenologies once its fifth and sixth power involvement are considered. Nevertheless, this provides a sign of the value of catastrophe-QSAR for achieving a deeper understanding of the molecular mechanics of specific interactions when the normal multi-linear QSAR does not assign transport descriptors with much predictive power.
- The relative statistical power, as defined by Equation (8), does not always parallel the Pearson coefficient or the relative correlation factors, as is evident from Tables 5 and 6. However, because it includes more statistical information, we consider a model as relevant when it has greater individual output of this newly introduced statistical index. In particular, neither the linear nor the multilinear QSAR framework provides a good fit between the statistical correlation and the relative statistical power using the structural parameter combinations considered. Instead, parabolic catastrophe correlations, the *cusp and butterfly models*, are revealed to be quite relevant, in particular regarding the formation energy (H) for which they show the highest Pearson correlation and relative statistical power values in comparison with the other descriptors plugged into these models. Unfortunately, for the two-variable descriptor models of Table 6, no consistency was found between the highest Pearson value and the relative statistical power apart from a few degenerate cases of descriptors for the parabolic models where the highest relative statistical power value corresponds with the highest Pearson correlation. Note that for the degenerate cases of Table 6, when two mixed descriptors can be combined in two distinct ways, the working model is considered to have maximum relative statistical power.

However, because the two-fold aim of the present research is to find the best predictive model and the molecular mechanism of action for the given set of molecules, the statistical indices of Tables 5 and 6 are employed to compute the first- and second-order differences (or distances) in relative statistical power as described by Equations (12–15) of Section 3. They correspond to the inter-descriptor/inter-modeling paths of molecular actions, whose minimum values are identified according to the prescription of Equation (16).

Through this minimal relative statistical power path recipe, once the models and descriptors predicted to be on the forefront of the structure-action interaction are selected, they are then further filtered with the testing set to finally identify the best predictive model and reveal the mechanism of action by means of the structural descriptors considered.

In the present case of the HIV inhibitors in Table 4, the data computed from Tables 5 and 6 provide the results for Tables 7–9, to be discussed herein:

- Table 7: At the individual descriptor level, the cusp and butterfly models are very close to each other for Log P and the forming energy H, which is even more relevant for the hydrophobicity, because for the forming energy it transpires from Table 5 that the butterfly model practically reduces to the cusp model because the sixth contribution virtually vanishes. However, for the structural influence on polarizability (POL) the butterfly and swallow tail are the closest models. When one considers the hierarchy of the individual descriptors according to their QSAR-I models in Table 5 in terms of the reduction in relative statistical power
  (17a) Log P→H→POL

through combining it with the catastrophes involved in Table 7, one correspondingly obtains the evolution cycle of the models:

(17b) (…→)[Butterfly]→[Cusp]→[Butterfly]→[SwallowTail](→…)

- Table 8: When the second order distance difference is considered between the individual inter-modeling paths of Table 7, it can nevertheless be considered through the further variations of paths of Table 7. Also, the QSAR-I and the fold (F) catastrophe model intervene in changing the influence on specific interactions from POL to H. Therefore, by counting the minimum hierarchy of these paths, the distance ordering is obtained as follows:
  (18a) (LogP÷H)→(H÷POL)→(POL÷LogP)

which, remarkably, confirms the descriptors’ cycles of influence in accordance with the first order prescription of Equation (17a). However, a more detailed succession is recorded for the inter-model evolution:

(18b) (…→)[Butterfly]≅[SwallowTail]→[QSAR-I]≅[Fold]→[Cusp]≅[Butterfly](…→)

When comparing cycles (18b) with (17b), it seems that the QSAR-I and Fold models appear in (18b) at the second cycle after the first one is performed on the prescription of (17b). For this reason also, the direct second order inter-descriptor-inter-models analysis is undertaken, and the results are reported in Table 9, to be discussed hereafter.

- Table 9: Interestingly, in terms of the two structural descriptors, the QSAR model is present even though its individual statistics are not the highest in Table 6; however, judging by the ordering of minimum paths recorded, the coupling descriptors hierarchy is established as:
  (19a) (H&POL)→(POL&Log   P)→(Log   P&H)

which is associated with the models’ evolution

(19b) (…→)[PU]→[EU]→[HU]→[QSAR](→[PU]…→)

One should make “contact” between the descriptor hierarchies [(17a), (18a), (19a)] and the models’ cycles [(17b), (18b) and (19b)] by means of the predictivity powers of the models along the minimum paths identified in Tables 7 and 9 with the single and double descriptors, respectively, for the non-Gaussian (NG) molecules of Table 4 and Figure 1. The results are systematically presented in Tables 10 and 11.

The results of correlation tests in Table 10 indicate the structure index–model activity hierarchy:

(20) $$|Y_{B}^{LogP}\rangle >|Y_{B}^{POL}\rangle >|Y_{C}^{LogP}\rangle >|Y_{ST}^{POL}\rangle >|Y_{C}^{H}\rangle >|Y_{B}^{H}\rangle$$

Somehow the influences of POL and H are reversed relative to the prescription by trial succession of Equation (17a), revealing hydrophobicity as the main influential factor. However, due to the fact that the predicted activities of POL in Table 10 are all in the “opposite evolution direction” with respect to the activities recorded in Table 4, *i.e.*, they are all negative, the uni-parametric tests and their associated hierarchy (20) are discarded, and one looks toward the second class of QSAR and catastrophe algorithms.

Instead, the test correlations of Table 11 provide the structure-activity ordering for the bi-parameter-models

(21) $$|Y_{II}^{LogP,H}\rangle >|Y_{HU}^{LogP,POL}\rangle >|Y_{HU}^{LogP,H}\rangle >{|Y_{PU}^{POL,H}\rangle }_{B}>{|Y_{EU}^{LogP,POL}\rangle }_{A}>{|Y_{EU}^{POL,H}\rangle }_{A}$$

**Figure 2:**
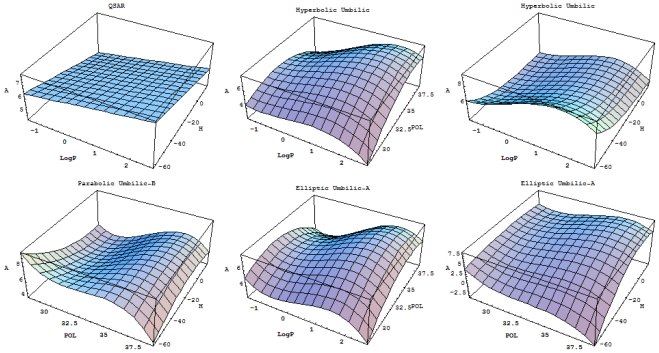
3D-representations of the QSAR and catastrophe activities for the tested models of Table 11 in the range of the structural indicators (Log P, Pol, H) as abstracted from Table 4.

**Figure 3:**
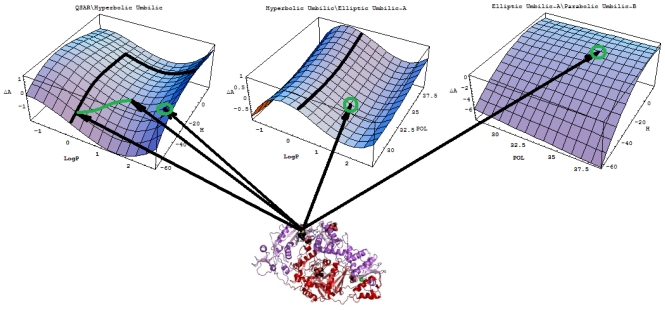
Determination of the structural domains of pyridinone-derivative type non-nucleoside reverse transcriptase inhibitors in the same range of structural descriptors by employing the principles of hydrophobicity, minimum polarizability, binding energy, and the minimum difference between the polynomial activity models of Figure 2; the hydrophobic pocket was identified in the p66 subunit of HIV-1-rt of specific transferase R221239 [48,49].

Remarkably, the hierarchy (21) starts with the QSAR model, which is revealed to be at the top of the validated catastrophe models with statistical performance even higher than through the predicted equation of Table 6 and the trial set of Table 4. Moreover, the QSAR-II model involves parameters (Log P & H) that are followed by the hyperbolic umbilic (HU) model in terms of (Log P & POL) parameters, in this way recovering the original mono-structural influences as anticipated by Equations (17a) and (18a). Thus, the series of models in Equation (21) is validated, and it will be further employed to establish the models’ successions and the molecular structural pattern of inhibiting anti-HIV-1 drug resistance. To this end, apart from the first and last models of Equation (21), which are associated with the maximum (0.778) and minimum (0.057) test performance, the middle catastrophe models provide closely related performance in the range (0.431, 0.468). Their graphical 3D-representation of the parametric domains Log P: (−1.50, 2.72), POL: (27.87, 38.48) and H: (−63.299, 17.808) of all (trial and test) structures in Table 4 are displayed in Figure 2. Next, it is apparent that they can be coupled according to the same spanned domains, thus forming the activity models’ differences $|Y_{II}^{LogP,H}\rangle -|Y_{HU}^{LogP,H}\rangle ,|Y_{HU}^{LogP,POL}\rangle -{|Y_{EU}^{LogP,POL}\rangle }_{A},{|Y_{EU}^{POL,H}\rangle }_{A}-{|Y_{PU}^{POL,H}\rangle }_{B}$, plotted in the top of Figure 3. Through registering the parameters and the models’ successions:

(22) $$\left[QSAR]\overset{LogP,H}{\to }[HU]\overset{LogP,POL}{\to }[EU]\overset{POL,H}{\to }[PU\right]$$

one may reach the following important conceptual-computational conclusions:

- The HIV-1 inhibitory activity is triggered by a hydrophobic interaction followed by energetic stabilization of the ligand/substrate (pyrididone derivative/viral protein) interaction here modeled by the heat of molecular formation and eventually completed by the ionic field influence herein represented by the polarizability descriptor.
- Although the QSAR multi-linear model should not be excluded from the molecular modeling of complex bio-chemical interactions, it should be complemented with other polynomial correlational catastrophe-type models that produce significant results comparable to those of other 3D-modeling procedures such as docking-based comparative molecular field analysis (CoMFA) and comparative molecular similarity indices analysis (CoMSIA) [24].

However, the issue remains of establishing the molecular structure most suitable for HIV-1 inhibitory activity among the considered pool of pyridinone derivatives in Table 4. To this end, the representations in Figure 3 are synergistically employed to identify the molecular structural domains that optimally promote binding of the pyridine derivative to the hydrophobic pocket in the p66 subunit of HIV-1 through searching for joint fulfillment of the following structural parameters and inter-model evolutionary generic principles:

- Log P: For positive values, the compound behaves hydrophobically and requires dissolution in an organic solvent; by contrast, for negative values the compound is hydrophilic and can be dissolved directly in an aqueous buffer. For Log P equal to 0, the compound partitions at a 1:1 organic-to-aqueous phase ratio, meaning that it is likely soluble in both organic and aqueous solvents and in cellular environments; thus, values of Log P equal to or greater than zero are selected to achieve hydrophobicity and suitability for the cellular environment [43,44], while characterizing the stacking bonding of aromatic rings [45];
- H: Because the formation of a compound from its elements usually is an exothermic process, most heats of formation are negative, and this is also a characteristic of the dynamic equilibrium of ligand-substrate interactions [46]; note that the advantage of using heat of formation as QSAR descriptor resides in the following: it thermodynamically relates with the free energy Δ*G*= −*RT*ln*K_eq_* by the equilibrium constant *_eq_* *K* which parallels the recorded activity at thermodynamic level [24]; it nevertheless expands the Gibbs free energy from the hydrogen to covalent bonding strength [45];
- PO: It is expected that “the natural direction of evolution of any system is towards a state of minimum polarizability” [47], while accounting for the dipolar interaction [45];
- Activity Models: Represent the same chemical-biological process providing their differences with respect to structural domains are minimized to zero.

These principles are applied to the activity models’ differences at the top of Figure 3, and they lead to the identification of the structural domain (and even points) characteristic of the pyridinone derivative most well-adapted to inhibiting the HIV-1 life cycle. The graphical results in Figure 3 suggest that the ordering of the structural indicators is:

(23a) $$|Y_{II}^{LogP,H}\rangle -|Y_{HU}^{LogP,H}\rangle :\{\text{Log P}:(0,1.5)\&\text{H}:(-55,-40)\;\text{kcal}/\text{mol}\}\cup \{\text{Log P}\approx 2.5\&\text{H}\approx -40\;\text{kcal}/\text{mol}\}$$

(23b) $$|Y_{HU}^{LogP,POL}\rangle -{|Y_{EU}^{LogP,POL}\rangle }_{A}:\text{Log P}\approx 1\&\text{POL}\approx 32\;{\overset{´}{Å}}^{3}$$

(23c) $${|Y_{EU}^{POL,H}\rangle }_{A}-{|Y_{PU}^{POL,H}\rangle }_{B}:\text{POL}\approx 34.5\;{\overset{´}{Å}}^{3};\text{H}\approx -10\;\text{kcal}/\text{mol}$$

The “solution” of system (23) gives the actual molecules in Table 4 predicted to be the most potent binding inhibitors, namely compounds **27** (Log P ≈ 2.72, H ≈ −39.459 kcal/mol, POL ≈ 35.55Ǻ^3^), **28** (Log P ≈ 1.06, H ≈ −34.478kcal/mol, POL ≈ 34.88Ǻ^3^), and **29** (Log P ≈ 0.96, H ≈ −21.361 kcal/mol, POL ≈ 35.17Ǻ^3^). Most impressively, these molecules were also predicted by the much more sophisticated methods of CoMFA and CoMSIA as having increased binding affinity between the aromatic ring (or wing 2 of the pyridinone derivative) and amino acid Tyr181 of the first molecule and Tyr188 of the last two. These two amino acids are very important in the inhibition of RT by NNRTIs because the most common mutations are Tyr181Cys and Tyr188Cys, and they are responsible for the emergence of viruses resistant to pyridinone derivatives. Therefore, designing pyridinone compounds that allow aromatic ring stacking interactions with Tyr181 and Tyr 188 may prevent these mutations and increase the activity of these anti-HIV drugs.

Overall, the QSAR presented here combined with catastrophe polynomial structure activity relationships provides a reliable conceptual and computational tool for identifying the mechanisms underlying ligand-subtract interactions and the structural domains best able to promote them. Consequently, this method should be further integrated into automated data processing and tested on other complex open systems with bio- or eco-toxicological relevance, especially where evolutionary life-cycles are present.

## 5. Conclusions

One of the most challenging battlefields in metabolic virology focuses on the complete and sustained inhibition of the HIV life cycle at its various levels. Thus: “an ideal anti-HIV agent should stop the virus’ progress and also the infection of healthy host cells, with no toxicity against normal cell physiology” [50]. Moreover, the ideal anti-HIV agent should avoid the drug-resistance phenomenon of HIV mutant variants. QSAR techniques are cost-effective computer-assisted drug design methods that can be used to obtain potential anti-HIV compounds with powerful biological effects and the lowest possible levels of side-effects and toxicity.

As the predictive roles of modeling and quantitative-structure-activity relationships (QSAR) in medicinal chemistry and drug synthesis are now recognized [51,52], thereby corroborating recent intriguing reports on the modest performance of direct statistical multilinear correlations in genotoxic carcinogenesis modeling of covalent drug binding to DNA followed by mutagenesis [53], the present study advances the idea of non-linear polynomial fits of observed/experimentally available *Activity* = *f* (*X*_1_, *X*_2_), with *X*_1_, *X*_2_ being structural physicochemical parameters (usually hydrophobicity, polarizability and/or forming heat energy in accordance with the basic recommendation of Hansch) [54] under the seven polynomial forms inspired by Thom’s catastrophe theory [1] (see Table 3).

As an application of the emerging *catastrophe-QSAR* analysis to a recently reported set of pyridinone derivatives with non-nucleoside reverse transcriptase inhibitor activity, [24] all the modeling stages required by the OECD-QSAR principles [32] are implemented here in a synergistic manner, namely:

- *A defined endpoint*: The hydrophobic binding of the inhibitor in the pocket of the p66 subunit of reverse-transcriptase was confirmed herein through the identification of hydrophobicity as the major influence among all the mono-nonlinear catastrophes employed; see Equation (17).
- *An unambiguous algorithm*: The Spectral-SAR minimum path principle [31,55–57] is here generalized to include relevant combination of statistical information (e.g., the correlation factor *R*, Student’s *t*-test, Fischer’s *F*-test) to provide an equal footing multi-dimensional Euler distance [see Equations (8–16)], thus avoiding the previously identified discrepancy in judging the mid-range performance in terms of correlation or other statistical factors [56].
- *A defined domain of applicability*: By performing linear *vs.* non-linear QSARs, the present strategy allows for the identification of recommended applicable structural domains through setting their difference to zero via inter-model activity minimization, which is equivalent to assuring the “smoothness” of the inhibitor-protein binding evolution towards the final steric inhibition output.
- *Appropriate measures of goodness-of-fit*, *robustness and predictivity*: The trial results were evaluated by external validation employing a testing set, which was selected by means of Gaussian *vs.* non-Gaussian distributions of the compounds’ activities, an improvement over the earlier arbitrariness of sampling the compounds only within a certain activity range. For instance, for linear QSAR the predicted correlation was superior to the tested correlation, thus confirming the reliability of this validation technique.
- *A mechanistic interpretation*: The selected succession of catastrophe-QSARs indicates that the inhibitor-HIV protein binding mutations that are involved in “birth and death” processes are associated with “waves” of induced activity in certain structural domain variants (see Figure 2). Moreover, the flat QSAR hypersurface should be complemented with catastrophe analysis to determine the specific structural domains for optimum interactions (see Figure 3) and for the associated molecular structure design of NNRT inhibitors.

Because the catastrophe-QSAR approach was found to successfully identify the molecular compounds with the most anti-HIV-1 potency as predicted by other 3D-QSAR methods, these results encourage further applications and implementations of Thom’s non-linear correlations with the goal of analytically modeling complex dynamic ligand-receptor interactions, especially on the molecular fragment or structural alert level [41], on a chemometric basis.

## Figures and Tables

**Table 1:** The QSAR working table for Equation (1) in the presence of M-structural descriptors for *N*-compounds with known activities.

Observed Activity	Structural		Predictor	Variables	
*A*	*X_1_*	…	*X_k_*	…	*X_M_*
*A*_1_	*x*_11_	…	*x*_1_*_k_*	…	*x*_1_*_M_*
*A*_2_	*x*_21_	…	*x*_2_*_k_*	…	*x*_2_*_M_*
⋮	⋮	⋮	⋮	⋮	⋮
*A_N_*	*x_N_*_1_	…	*x_Nk_*	…	*x_NM_*

**Table 2:** Thom’s Classification of Elementary Catastrophes [36,37].

Name	Co-dimension	Co-rank	Universal unfolding	Parametric Representation
Fold	1	1	*x*^3^ + *ux*	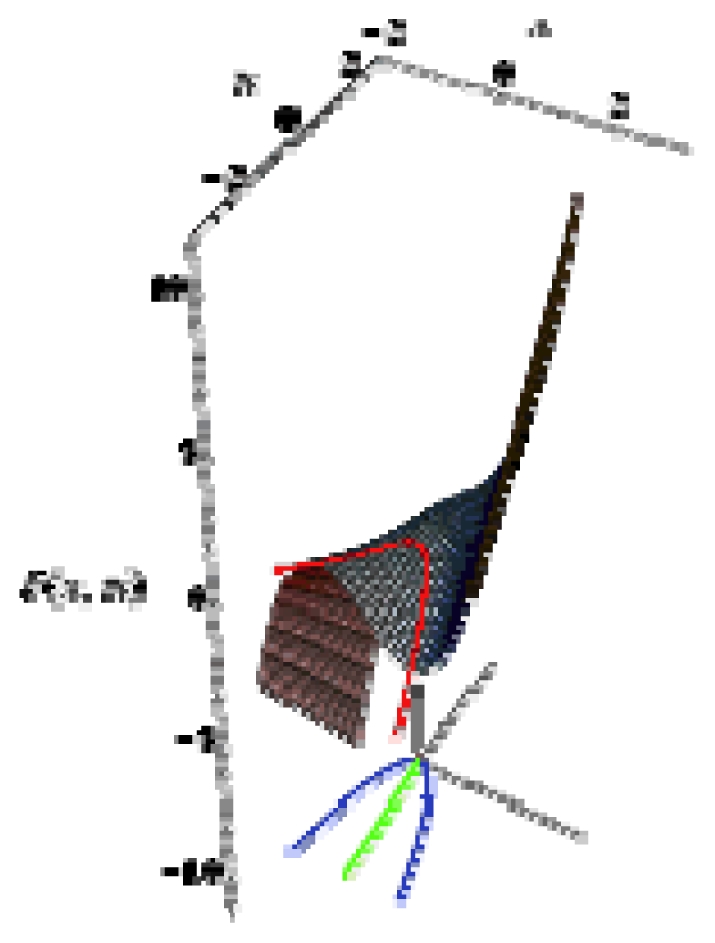
Cusp	2	1	*x*^4^ + *ux*^2^ + *vx*	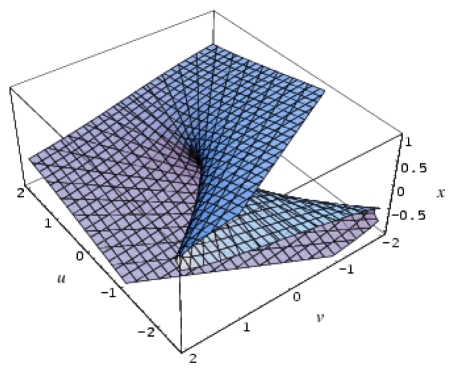
Swallow tail	3	1	*x*^5^ + *ux*^3^ + *vx*^2^ + *wx*	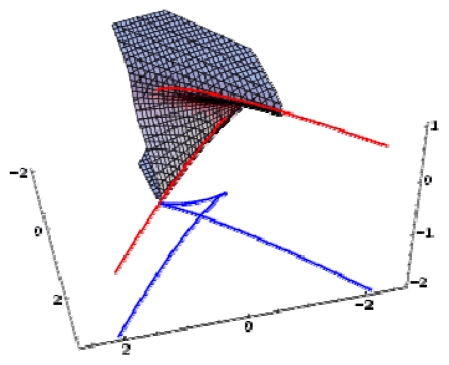
Butterfly	4	1	*x*^6^ + *ux*^4^ + *vx*^3^ + *wx*^2^ + *tx*	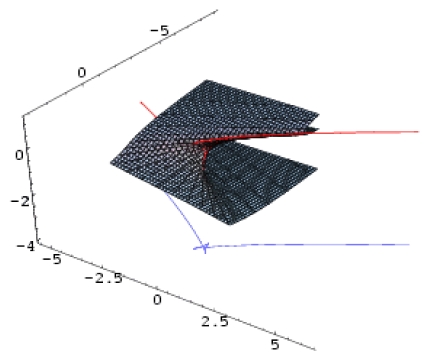
Hyperbolic umbilic	3	2	*x*^3^ + *y*^3^ + *uxy* + *vx* + *wy*	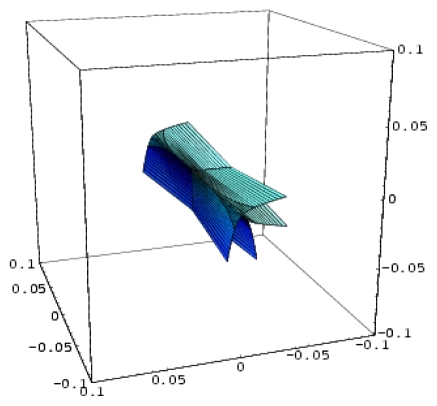
Elliptic umbilic	3	2	*x*^3^ − *xy*^2^ + *u*(*x*^2^ + *y*^2^ ) + *vx* + *wy*	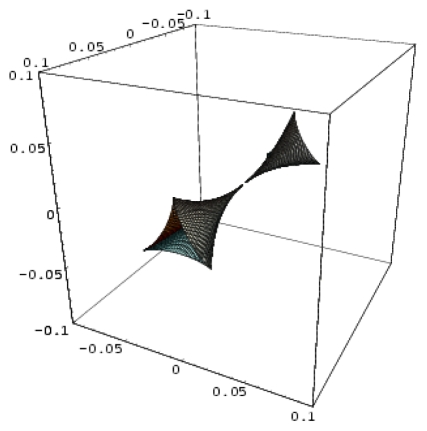
Parabolic umbilic	4	2	*x*^2^*y* + *y*^4^ + *ux*^2^ + *vy*^2^ + *wx* + *ty*	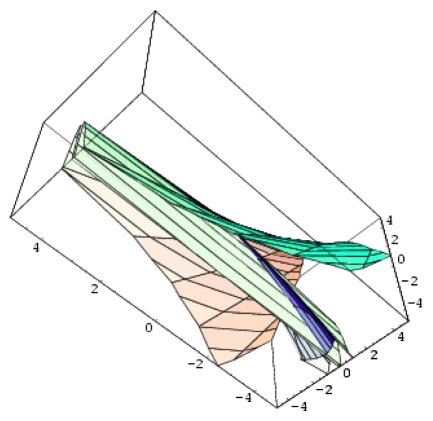

**Table 3:** Algebraic realization of Thom’s elementary catastrophes as uni- and bi- nonlinear QSARs. The systematics of the sub-indices indicate consecutive coupled pairs, where each pair is interpreted as: the index of a structural factor followed by its power.

Model	QSAR Equation
**GROUP I**: with one descriptor only, |*X*_1_〉	

QSAR-(I)	$|Y_{I}\rangle =a_{0}|1\rangle +a_{11}|X_{1}\rangle$
Fold	$|Y_{F}\rangle =f_{0}|1\rangle +f_{11}|X_{1}\rangle +f_{13}|X_{1}^{3}\rangle$
Cusp	$|Y_{C}\rangle =c_{0}|1\rangle +c_{11}|X_{1}\rangle +c_{12}|X_{1}^{2}\rangle +c_{14}|X_{1}^{4}\rangle$
Swallow tail	$|Y_{ST}\rangle =s_{0}|1\rangle +s_{11}|X_{1}\rangle +s_{12}|X_{1}^{2}\rangle +s_{13}|X_{1}^{3}\rangle +s_{15}|X_{1}^{5}\rangle$
Butterfly	$|Y_{B}\rangle =b_{0}|1\rangle +b_{11}|X_{1}\rangle +b_{12}|X_{1}^{2}\rangle +b_{13}|X_{1}^{3}\rangle +b_{14}|X_{1}^{4}\rangle +b_{16}|X_{1}^{6}\rangle$

**GROUP II**: with two descriptors, |*X*_1_〉,|*X*_2_〉	

QSAR- (II)	$|Y_{II}\rangle =q_{0}|1\rangle +q_{11}|X_{1}\rangle +q_{21}|X_{2}\rangle$
Hyperbolic umbilic	$|Y_{HU}\rangle =h_{0}|1\rangle +h_{11}|X_{1}\rangle +h_{21}|X_{2}\rangle +h_{1121}|X_{1}X_{2}\rangle +h_{13}|X_{1}^{3}\rangle +h_{23}|X_{2}^{3}\rangle$
Elliptic umbilic	$|Y_{EU}\rangle =e_{0}|1\rangle +e_{11}|X_{1}\rangle +e_{21}|X_{2}\rangle +e_{12}|X_{1}^{2}\rangle +e_{22}|X_{2}^{2}\rangle +e_{1122}|X_{1}X_{2}^{2}\rangle +e_{13}|X_{1}^{3}\rangle$
Parabolic umbilic	$|Y_{PU}\rangle =p_{0}|1\rangle +p_{11}|X_{1}\rangle +p_{21}|X_{2}\rangle +p_{12}|X_{1}^{2}\rangle +p_{22}|X_{2}^{2}\rangle +p_{1221}|X_{1}^{2}X_{2}\rangle +p_{24}|X_{2}^{4}\rangle$

**Table 4:** Actual working reverse transcriptase pyridinone inhibitors grouped in Gaussian (G) and non-Gaussian (NG) molecular congeneric sets with their structural information (hydrophobicity, Log P; molecular polarizability POL [Å^3^] and total optimized energy of formation H [kcal/mol]) computed upon the semi-empirical PM3 method [42], along with their observed activity A = Log (1/IC50) [24].

No.	Type	WORKING MOLECULES		A^obs^	QSAR parameters		
		Structure	Name	Log (1/IC_50_)	Log P	POL (Å^3^)	H (kcal/mol)
1.	G1	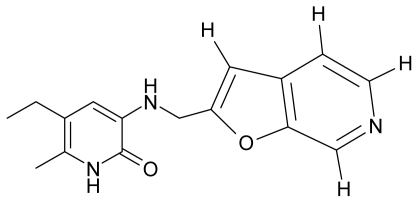	3-{[(6′-azabenzofuran-2′-yl) methyl]amino}-5-ethyl-6-methylpyridin-2(1H)-one	3.98	−0.54	31.21	−14.67
2.	G2	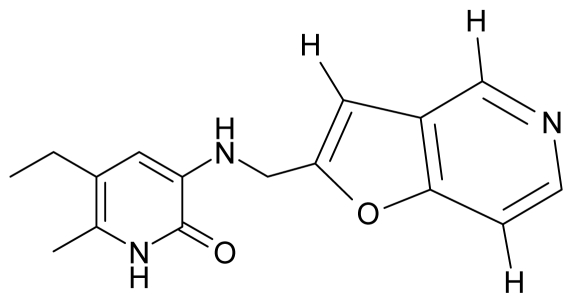	3-{[(5′-azabenzofuran-2′-yl) methyl]amino}-5-ethyl-6-methylpyridin-2(1H)-one	4.49	−0.54	31.21	−16.195
3.	G3	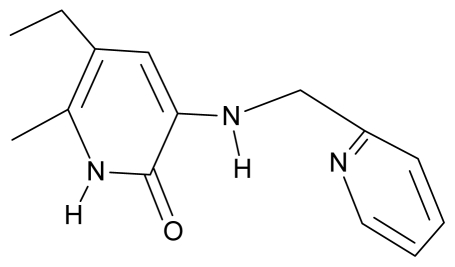	3-{[(pyridine-2′-yl) methyl]amino}-5-ethyl-6-methylpyridin-2(1H)-one	4.82	0.21	27.87	-5.854
4.	G4	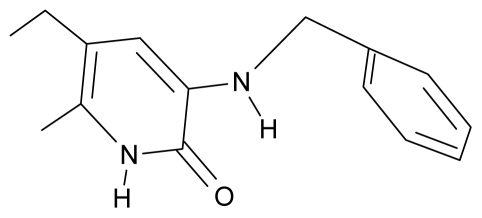	3-benzylamino-5-ethyl-6-methylpyridin-2(1H)-one	5.27	0.67	28.58	−11.659
5.	G5	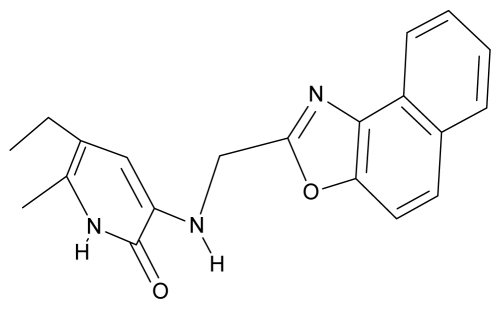	3-{[(1′,3′-naftoxazol-2′-yl) methyl]amino}-5-ethyl-6-methylpyridin-2(1H)-one	5.57	1.20	38.48	−1.878
6.	G6	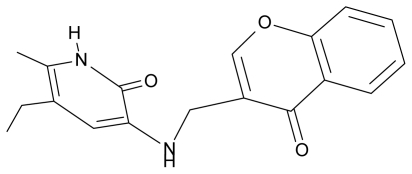	3-{[(1′-benzopyran-4′-one-3′-yl) methyl]amino}-5-ethyl-6-methylpyridin-2(1H)-one	5.96	−0.71	33.84	−61.455
7.	G7	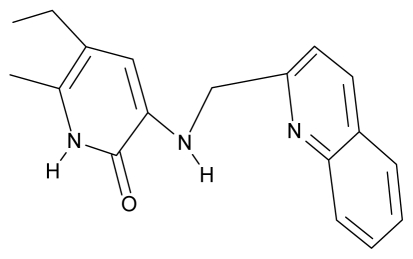	3-{[(benzopyridine-2′-yl) methyl]amino}-5-ethyl-6-methylpyridin-2(1H)-one	6.28	1.16	35.14	11.246
8.	G8	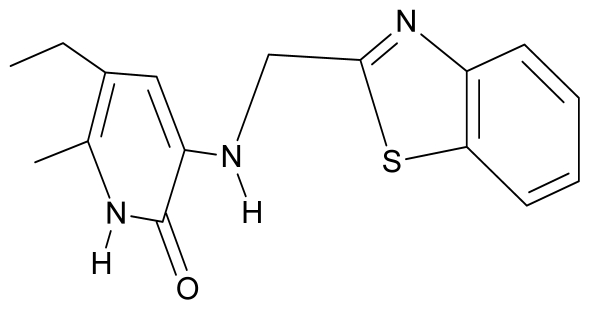	3-{[(1′,3′-benzothiazole-2′-yl) methyl]amino}-5-ethyl-6-methylpyridin-2(1H)-one	6.46	0.54	33.57	17.808
9.	G9	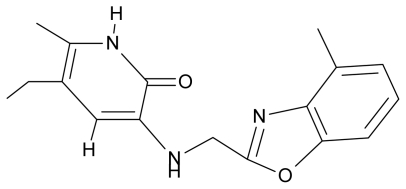	3-{[(4′-methylbenzoxazole-2′-yl) methyl]amino}-5-ethyl-6-methylpyridin-2(1H)-one	6.92	0.67	33.05	−27.613
10.	G10	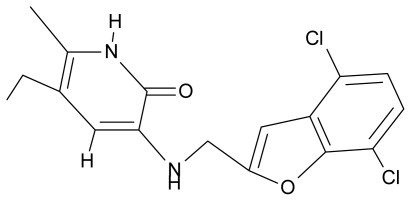	3-{[(4′,7′-dichlorobenzofuran-2′-yl) methyl]amino}-5-ethyl-6-methylpyridin-2(1H)-one	7.24	0.88	35.78	−33.749
11.	G11	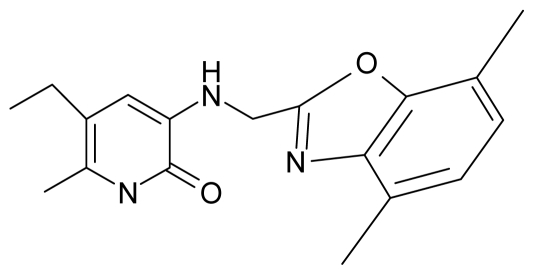	3-{[(4′,7′-dimethylbenzoxazol-2′-yl) methyl]amino}-5-ethyl-6-methylpyridin-2(1H)-one	7.7	1.13	34.88	−38.048
12.	G12	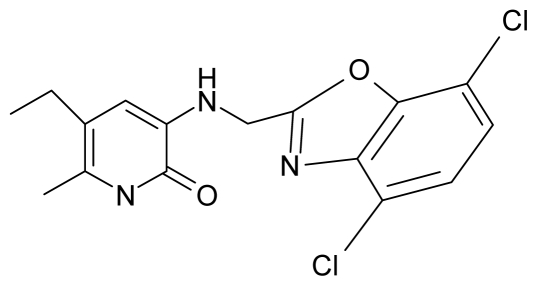	3-{[(4′,7′-dichlorobenzoxazol-2′-yl) methyl]amino}-5-ethyl-6-methylpyridin-2(1H)-one	7.72	1.24	35.07	−30.071
13.	G13	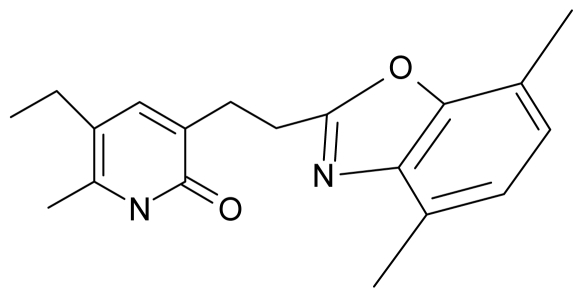	3-[(4′,7′-dimethylbenzoxazol-2′-yl) ethyl]-5-ethyl-6-methylpyridin-2(1H)-one	7.55	2.62	35.37	−47.701
14.	G14	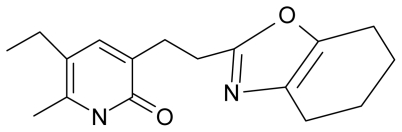	3-[(4′,5′,6′,7′-tetrahydrobenzoxazole-2′-yl) ethyl]-5-ethyl-6-methylpyridin-2(1H)-one	7.24	−0.02	32.08	−63.299
15.	G15	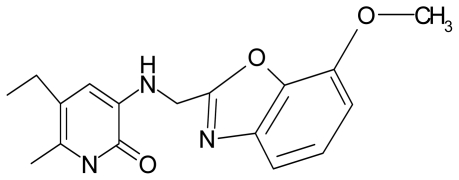	3-{[(4′-methoxybenzoxazole-2′-yl) methyl]amino}-5-ethyl-6-methylpyridin-2(1H)-one	6.74	−0.05	33.68	−54.452
16.	G16	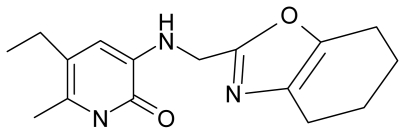	3-[(4′,5′,6′,7′-tetrahydrobenzoxazole-2′-yl) methyl]amino}-5-ethyl-6-methylpyridin-2(1H)-one	6.55	−1.50	31.59	−50.643
17.	G17	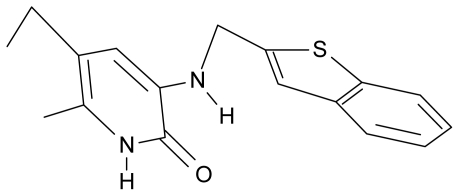	3-{[(benzothiophene-2′-yl) methyl] amino}-5-ethyl-6-methylpyridin-2(1H)-one	6.30	0.19	34.28	11.703
18.	G18	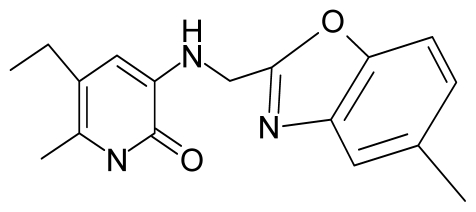	3-{[(5′-methylbenzoxazole-2′-yl) methyl]amino}-5-ethyl-6-methylpyridin-2(1H)-one	5.90	0.67	33.05	−27.741
19.	G19	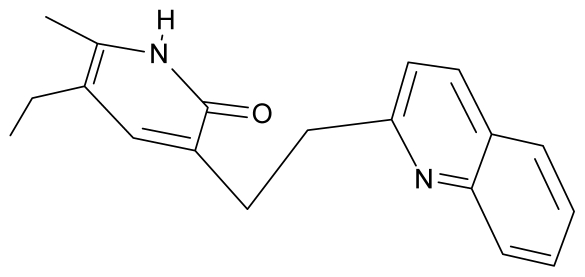	3-[(benzopyridine-2′-yl) ethyl]5-ethyl-6-methylpyridin-2(1H)-one	5.61	2.71	35.62	3.331
20.	G20	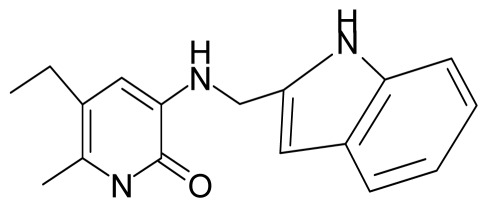	3-{[(indol-2′-yl) methyl] amino}-5-ethyl-6-methylpyridin-2(1H)-one	5.36	−0.34	32.63	4.727
21.	G21	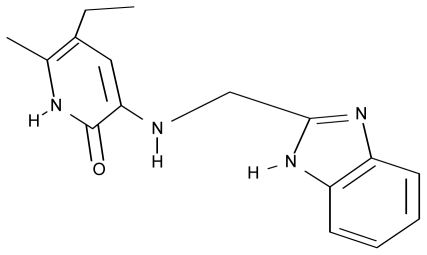	3-{[(quinazolin-2′-yl) methyl]amino}-5-ethyl-6-methylpyridin-2(1H)-one	5.12	0.02	31.92	8.171
22.	G22	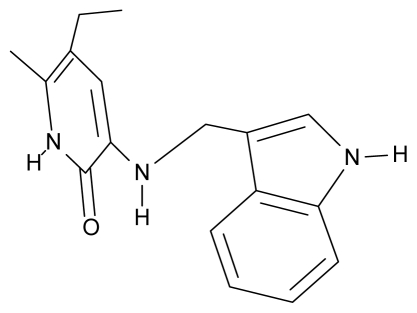	3-{[(indol-3′-yl)methyl] amino}-5-ethyl-6-methylpyridin-2(1H)-one	4.65	−0.43	32.63	2.957
23.	G23	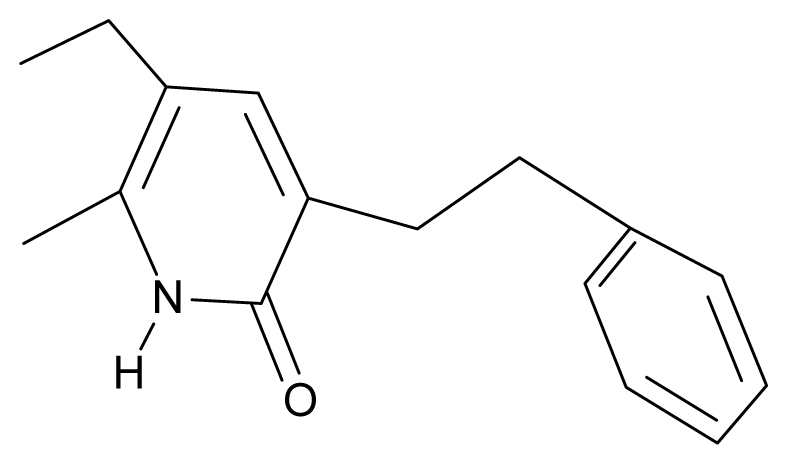	3-(β-phenilethyl)-5-ethyl-6-methylpyridin-2(1H)-one	4.30	2.36	29.06	−23.245
24.	NG1	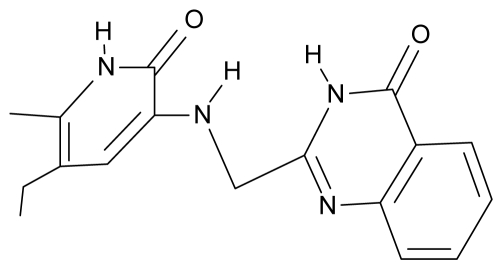	3-{[(4′-quinozolone-2′-yl) methyl]amino}-5-ethyl-6-methylpyridin-2(1H)-one	5.60	−0.47	33.85	−36.959
25.	NG2	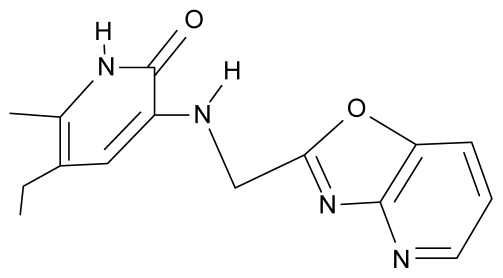	3-{[(3′,4′-diazobenzofuran-2′-yl) methyl]amino}-5-ethyl-6-methylpyridin-2(1H)-one	5.72	0.05	30.50	−8.120
26.	NG3	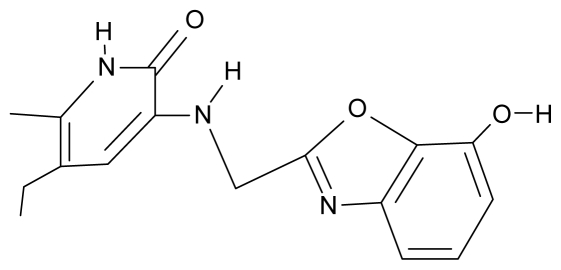	3-{[(7′-hydroxybenzoxazole-2′-yl) methyl]amino}-5-ethyl-6-methylpyridin-2(1H)-one	6.36	−0.08	31.85	−62.189
27.	NG4	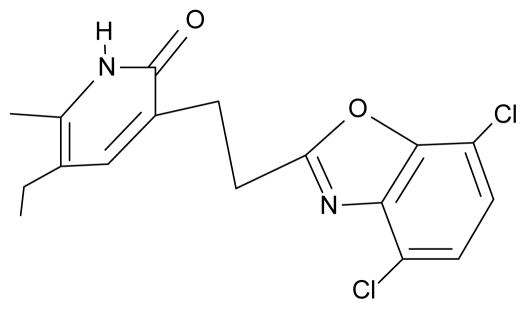	3-[(4′,7′-dichlorobenzoxazole-2′-yl) ethyl]-5-ethyl-6-methylpyridin-2(1H)-one	7.85	2.72	35.55	−39.459
28.	NG5	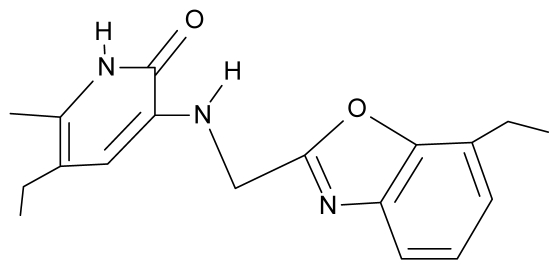	3-{[(7′-ethylbenzoxazole-2′-yl) methyl]amino}-5-ethyl-6-methylpyridin-2(1H)-one	6.59	1.06	34.88	−34.478
29.	NG6	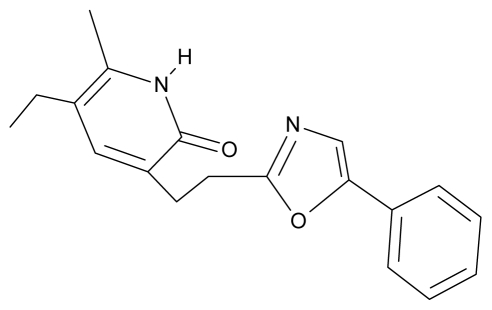	3-[(5′-phenyl-oxazole-2′-yl) ethyl]-5-ethyl-6-methylpyridin-2(1H)-one	6.41	0.96	35.17	−21.361
30.	NG7	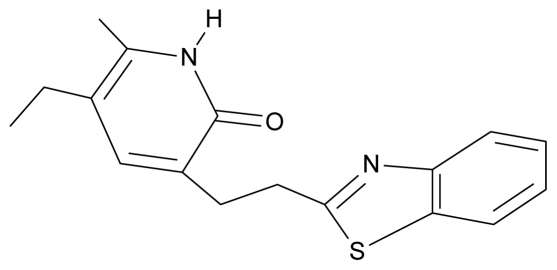	3-[(benzothiazole-2′-yl) ethyl]-5-ethyl-6-methylpyridin-2(1H)-one	6.43	2.02	34.06	8.873
31.	NG8	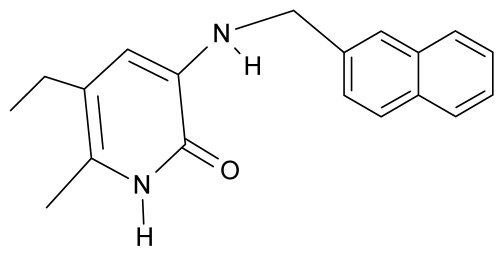	3-{[(2′naphtyl) methyl] amino}-5-ethyl-6-methylpyridin-2(1H)-one	6.34	1.67	35.85	5.495
32.	NG9	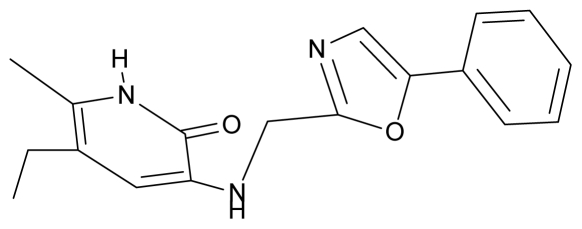	3-{[(5′-phenyl-oxazole-2′-yl) methyl]amino}-5-ethyl-6-methylpyridin-2(1H)-one	5.63	−0.53	34.69	−10.850

**Table 5:** Correlation equations for the Group-I models of Table 3 and the molecular structures and data of Table 4.

Catastrophe	QSAR Model	*R*_Pearson_(a)	*R*_ALG_(b)	*r*(c)	*t-Stud.*	*t*(d)	*Fisher*	*f*(e)	Π(f)
QSAR (I)	$|Y_{I}^{LogP}\rangle =5.861|1\rangle +0.240|LogP\rangle$	0.228	0.984	4.317	22.344	7.854	1.150	0.143	8.963
	$|Y_{I}^{POL}\rangle =-2.257|1\rangle +0.249|POL\rangle$	0.554	0.989	1.784	−0.832	−0.292	9.284	1.158	2.147
	$|Y_{I}^{H}\rangle =5.57|1\rangle -0.021|H\rangle$	0.476	0.987	2.074	20.597	7.24	6.156	0.768	7.57
Fold (F)	$|Y_{F}^{LogP}\rangle =5.854|1\rangle +0.738|LogP\rangle -0.106|LogP^{3}\rangle$	0.382	0.986	2.581	22.936	8.062	1.705	0.213	8.468
	$|Y_{F}^{POL}\rangle =-24.206|1\rangle +1.26|POL\rangle -3\cdot 10^{-4}|POL^{3}\rangle$	0.601	0.989	1.646	−1.422	−0.45	5.650	0.704	1.859
	$|Y_{F}^{H}\rangle =5.58|1\rangle -0.016|H\rangle -2\cdot 10^{-6}|H^{3}\rangle$	0.481	0.987	2.053	20.095	7.063	3.01	0.375	7.365
Cusp (C)	$|Y_{C}^{LogP}\rangle =5.707|1\rangle +0.426|LogP\rangle +0.372|LogP^{2}\rangle -0.071|LogP^{4}\rangle$	0.348	0.985	2.832	16.120	5.666	0.872	0.109	6.335
	$|Y_{C}^{POL}\rangle =431.26|1\rangle -35.694|POL\rangle +0.833|POL^{2}\rangle -10^{-4}|POL^{4}\rangle$	0.713	0.992	1.391	2.240	0.787	6.558	0.818	1.796
	$|Y_{C}^{H}\rangle =5.006|1\rangle +0.042|H\rangle +0.003|H^{2}\rangle -10^{-6}|H^{4}\rangle$	0.764	0.993	1.300	19.802	6.960	8.864	1.105	7.166
Swallow tail (ST)	$\begin{array}{l} |Y_{ST}^{LogP}\rangle =5.649|1\rangle +1.608|LogP\rangle +0.326|LogP^{2}\rangle \\ -0.978|LogP^{3}\rangle +0.0093|LogP^{5}\rangle \end{array}$	0.575	0.989	1.720	18.665	6.561	2.222	0.277	6.788
	$\begin{array}{l} |Y_{ST}^{POL}\rangle =1476.244|1\rangle -156.079|POL\rangle \\ +5.791|POL^{2}\rangle -0.079|POL^{3}\rangle +5.5\cdot 10^{-6}|POL^{5}\rangle \end{array}$	0.715	0.992	1.387	0.45	0.158	4.708	0.587	1.515
	$\begin{array}{l} |Y_{ST}^{H}\rangle =4.884|1\rangle +0.031|H\rangle +0.004|H^{2}\rangle \\ +5.2\cdot 10^{-5}|H^{3}\rangle +4\cdot 10^{-10}|H^{5}\rangle \end{array}$	0.763	0.993	1.302	15.608	5.486	6.263	0.781	5.692
Butterfly (B)	$\begin{array}{l} |Y_{B}^{LogP}\rangle =5.646|1\rangle +1.464|LogP\rangle +0.303|LogP^{2}\rangle \\ -0.688|LogP^{3}\rangle -0.041|LogP^{4}\rangle +0.027|LogP^{6}\rangle \end{array}$	0.578	0.989	1.711	15.169	5.332	1.704	0.212	5.604
	$\begin{array}{l} |Y_{B}^{POL}\rangle =-16485.827|1\rangle +2491.049|POL\rangle -146.094|POL^{2}\rangle \\ +4.037|POL^{3}\rangle -0.047|POL^{4}\rangle +2.9\cdot 10^{-6}|POL^{6}\rangle \end{array}$	0.718	0.992	1.382	−0.355	−0.125	3.619	0.451	1.459
	$\begin{array}{l} |Y_{B}^{H}\rangle =4.876|1\rangle +0.110|H\rangle +0.004|H^{2}\rangle \\ -2.3\cdot 10^{-4}|H^{3}\rangle -7.67\cdot 10^{-6}|H^{4}\rangle +6.3\cdot 10^{-10}|H^{6}\rangle \end{array}$	0.856	0.996	1.163	19.088	6.709	9.349	1.166	6.908

(a) the statistical Pearson correlation factor;

(b) computed from Equation (7);

(c) computed from Equation (9);

(d) computed from Equation (10) with $t_{\begin{array}{l} Tabulated\\ \left(0.99;20\right) \end{array}}=2.845$;

(e) computed from Equation (11) with $F_{\begin{array}{l} Tabulated\\ \left(0.99;1,21\right) \end{array}}=8.02$;

(f) computed from Equation (8).

**Table 6:** Correlation equations for the Group-II models of Table 3 and the molecular structures and data of Table 4.

Catastrophe	QSAR Model	R_Pearson_(a)	R_ALG_(b)	*r*(c)	*t-Stud.*	*t*(d)	*Fisher*	*f*(e)	Π(f)
QSAR (II)	$|Y_{II}^{LogP,POL}\rangle =-2.044|1\rangle +0.051|LogP\rangle +0.242|POL\rangle$	0.556	0.989	1.778	−0.702	−0.245	4.464	0.763	1.9504
	$|Y_{II}^{LogP,H}\rangle =5.379|1\rangle +0.304|LogP\rangle -0.023|H\rangle$	0.556	0.989	1.778	18.564	6.489	4.468	0.764	6.771
	$|Y_{II}^{POL,H}\rangle =-2.637|1\rangle +0.248|POL\rangle -0.021|H\rangle$	0.728	0.992	1.363	−1.151	−0.402	11.302	1.932	2.398
Hyperbolic umbilic (HU)	$\begin{array}{l} |Y_{HU}^{LogP,POL}\rangle =-39.499|1\rangle -2.463|LogP\rangle +2.043|POL\rangle \\ +0.104|\left(LogP)(POL\right)\rangle -0.145|LogP^{3}\rangle -6\cdot 10^{-4}|POL^{3}\rangle \end{array}$	0.715	0.992	1.387	−2.215	−0.774	3.561	0.609	1.701
	$\begin{array}{l} |Y_{HU}^{LogP,H}\rangle =5.319|1\rangle +1.083|LogP\rangle -0.002|H\rangle \\ -0.003|\left(LogP)(H\right)\rangle -0.161|LogP^{3}\rangle -9\cdot 10^{-6}|H^{3}\rangle \end{array}$	0.736	0.992	1.3485	19.328	6.756	4.019	0.687	6.923
	$\begin{array}{l} |Y_{HU}^{POL,H}\rangle =-13.192|1\rangle +0.766|POL\rangle +0.122|H\rangle \\ -0.004|\left(POL)(H\right)\rangle -2\cdot 10^{-4}|POL^{3}\rangle -5.1\cdot 10^{-7}|H^{3}\rangle \end{array}$	0.755	0.993	1.315	−0.79	−0.276	4.503	0.770	1.549
Elliptic umbilic (EU)	$\begin{array}{l} {|Y_{EU}^{LogP,POL}\rangle }_{A}=-69.262|1\rangle -0.556|LogP\rangle +4.531|POL\rangle \\ +0.443|LogP^{2}\rangle -0.068|POL^{2}\rangle \\ +0.002|\left(LogP)(POL^{2}\right)\rangle -0.322|LogP^{3}\rangle \end{array}$	0.757	0.993	1.312	−2.548	−0.891	3.582	0.612	1.670
	$\begin{array}{l} {|Y_{EU}^{LogP,POL}\rangle }_{B}=644.623|1\rangle +0.022|LogP\rangle -59.934|POL\rangle \\ +0.467|LogP^{2}\rangle +1.855|POL^{2}\rangle \\ -0.015|\left(POL)(LogP^{2}\right)\rangle -0.019|POL^{3}\rangle \end{array}$	0.722	0.992	1.374	1.866	0.652	2.908	0.497	1.600
	$\begin{array}{l} {|Y_{EU}^{LogP,H}\rangle }_{A}=5.022|1\rangle +0.974|LogP\rangle +0.025|H\rangle \\ +0.530|LogP^{2}\rangle +0.001|H^{2}\rangle \\ +2.87\cdot 10^{-4}|\left(LogP)(H^{2}\right)\rangle -0.359|LogP^{3}\rangle \end{array}$	0.843	0.995	1.181	20.638	7.214	6.542	1.118	7.395
Elliptic umbilic (EU)	$\begin{array}{l} {|Y_{EU}^{LogP,H}\rangle }_{B}=4.779|1\rangle +0.643|LogP\rangle +0.029|H\rangle \\ -0.211|LogP^{2}\rangle +0.004|H^{2}\rangle \\ +0.001|\left(H)(LogP^{2}\right)\rangle +5\cdot 10^{-5}|H^{3}\rangle \end{array}$	0.851	0.995	1.170	17.047	5.958	7.015	1.199	6.189
	$\begin{array}{l} {|Y_{EU}^{POL,H}\rangle }_{A}=802.877|1\rangle -74.631|POL\rangle -0.02|H\rangle \\ +2.291|POL^{2}\rangle +0.005|H^{2}\rangle \\ -2\cdot 10^{-4}|\left(POL)(H^{2}\right)\rangle -0.023|POL^{3}\rangle \end{array}$	0.857	0.996	1.162	3.124	1.092	7.346	1.256	2.029
	$\begin{array}{l} {|Y_{EU}^{POL,H}\rangle }_{B}=11.888|1\rangle -0.562|POL\rangle +0.068|H\rangle \\ +0.011|POL^{2}\rangle +0.004|H^{2}\rangle \\ -4\cdot 10^{-5}|\left(H)(POL^{2}\right)\rangle +4\cdot 10^{-5}|H^{3}\rangle \end{array}$	0.853	0.996	1.167	0.532	0.186	7.120	1.217	1.696
Parabolic umbilic (PU)	$\begin{array}{l} {|Y_{PU}^{LogP,POL}\rangle }_{A}=474.915|1\rangle +0.021|LogP\rangle -39.256|POL\rangle \\ +0.454|LogP^{2}\rangle +0.914|POL^{2}\rangle \\ -0.015|(LogP^{2})\left(POL\right)\rangle -10^{-4}|POL^{4}\rangle \end{array}$	0.722	0.992	1.374	1.817	0.635	2.905	0.497	1.593
	$\begin{array}{l} {|Y_{PU}^{LogP,POL}\rangle }_{B}=-67.522|1\rangle -1.539|LogP\rangle +4.444|POL\rangle \\ +0.573|LogP^{2}\rangle -0.067|POL^{2}\rangle \\ +0.002|(POL^{2})\left(LogP\right)\rangle -0.115|LogP^{4}\rangle \end{array}$	0.703	0.992	1.411	−2.219	−0.776	2.611	0.446	1.671
Parabolic umbilic (PU)	$\begin{array}{l} {|Y_{PU}^{LogP,H}\rangle }_{A}=4.852|1\rangle +0.700|LogP\rangle +0.041|H\rangle \\ -0.240|LogP^{2}\rangle +0.004|H^{2}\rangle \\ +0.002|\left(LogP^{2}\right)(H)\rangle -10^{-6}|H^{4}\rangle \end{array}$	0.874	0.996	1.140	20.243	7.075	8.645	1.478	7.317
	$\begin{array}{l} {|Y_{PU}^{LogP,H}\rangle }_{B}=5.10|1\rangle +0.552|LogP\rangle +0.020|H\rangle \\ +0.460|LogP^{2}\rangle +9.57\cdot 10^{-4}|H^{2}\rangle \\ +1.93\cdot 10^{-4}|(H^{2})\left(LogP^{2}\right)\rangle -0.099|LogP^{4}\rangle \end{array}$	0.767	0.993	1.295	16.828	5.882	3.815	0.652	6.058
	$\begin{array}{l} {|Y_{PU}^{POL,H}\rangle }_{A}=8.876|1\rangle -0.366|POL\rangle +0.069|H\rangle \\ +0.008|POL^{2}\rangle +0.003|H^{2}\rangle \\ -3.7\cdot 10^{-5}|\left(POL^{2})(H\right)\rangle -4.5\cdot 10^{-7}|H^{4}\rangle \end{array}$	0.841	0.995	1.183	0.386	0.135	6.447	1.102	1.623
	$\begin{array}{l} {|Y_{PU}^{POL,H}\rangle }_{B}=595.212|1\rangle -48.906|POL\rangle -0.019|H\rangle \\ +1.129|POL^{2}\rangle +5\cdot 10^{-3}|H^{2}\rangle \\ -1.49\cdot 10^{-4}|\left(H^{2})(POL\right)\rangle -1.73\cdot 10^{-4}|POL^{4}\rangle \end{array}$	0.856	0.996	1.163	3.074	1.074	7.292	1.246	2.015

(a) the statistical Pearson correlation factor;

(b) computed from Equation (7);

(c) computed from Equation (9);

(d) computed from Equation (10) with $t_{\begin{array}{l} Tabulated\\ \left(0.99;19\right) \end{array}}=2.861$;

(e) computed from Equation (11) with $F_{\begin{array}{l} Tabulated\\ \left(0.99;2,20\right) \end{array}}=5.85$;

(f) computed from Equation (8).

**Table 7:** Single-structure matrices of the Euclidean distances ΔΠ*_I_* of the QSAR and catastrophe models’ relative statistics of Table 5 employing Equation (12).

Log P	*F*	*C*	*ST*	*B*
***QSAR***	1.750	2.645	2.905	3.627
***F***		2.411	1.732	2.865
***C***			1.437	1.174
***ST***				1.231

POL	*F*	*C*	*ST*	*B*
***QSAR***	0.517	1.198	0.828	0.830
***F***		1.317	0.717	0.524
***C***			0.670	0.983
***ST***				0.314

H	*F*	*C*	*ST*	*B*
***QSAR***	0.431	0.89	1.916	1.127
***F***		1.054	1.793	1.242
***C***			1.509	0.292
***ST***				1.29

**Table 8:** Differences Δ^2^Π*_I_* between the single-structure matrices of the Euclidean distances in Table 7.

|Log P ÷ POL|	*F*	*C*	*ST*	*B*
***QSAR***	1.233	1.446	2.076	2.797
***F***		1.094	1.015	2.341
***C***			0.767	0.191
***ST***				0.917

|Log P ÷ H|	*F*	*C*	*ST*	*B*
***QSAR***	1.32	1.755	0.988	2.501
***F***		1.358	0.062	1.624
***C***			0.072	0.882
***ST***				0.059

|POL ÷ H|	*F*	*C*	*ST*	*B*
***QSAR***	0.086	0.309	1.088	0.297
***F***		0.264	1.076	0.717
***C***			0.839	0.691
***ST***				0.976

**Table 9:** Single-structure matrices of the Euclidean distances ΔΠ*_II_* of the QSAR and catastrophe models’ relative statistics of Table 6 employing Equation (12); note that for the degenerate models of Table 6 that one is employed that displays higher relative statistical power ( Π).

Log P^POL	*HU*	*EU*	*PU*
***QSAR***	0.675	0.810	1.005
***HU***		0.139	1.414
***EU***			1.531

Log P^H	*HU*	*EU*	*PU*
***QSAR***	0.512	0.917	1.123
***HU***		0.964	0.878
***EU***			1.152

POL^H	*HU*	*EU*	*PU*
***QSAR***	1.170	1.652	1.640
***HU***		1.46	1.440
***EU***			0.02

**Table 10:** Predicted activity as computed for the non-Gaussian molecules of Table 4 with the models of Table 5 founded along the minimum paths of Table 7; for each predicted model, its correlation with the observed activity is indicated at the bottom of the table.

Model	$|Y_{C}^{LogP}\rangle$	$|Y_{C}^{H}\rangle$	$|Y_{ST}^{POL}\rangle$	$|Y_{B}^{LogP}\rangle$	$|Y_{B}^{POL}\rangle$	$|Y_{B}^{H}\rangle$
Molecule						
***NG1***	5.586	6.179	5.294	5.094	−20.595	5.687
***NG2***	5.729	4.885	4.294	5.719	−9.764	4.360
***NG3***	5.676	0.415	4.708	5.531	−13.457	−7.932
***NG4***	5.729	6.156	5.149	6.657	−29.709	5.259
***NG5***	6.487	6.141	5.309	6.705	−25.700	5.923
***NG6***	6.399	5.438	5.258	6.708	−27.365	5.219
***NG7***	6.903	5.631	5.319	5.311	−21.540	5.984
***NG8***	6.904	5.334	5.027	5.995	−31.693	5.566
***NG9***	5.580	4.9357	5.328	5.054	−24.666	4.383
***R-Pearson***	***0.195***	***0.129***	***0.174***	***0.701***	***0.488***	***0.026***

**Table 11:** Predicted activity as computed for the non-Gaussian molecules of Table 4 with the models of Table 6 founded along the minimum paths of Table 9; for each predicted model, its correlation with the observed activity is indicated at the bottom of the table.

Model	$|Y_{II}^{LogP,H}\rangle$	$|Y_{HU}^{LogP,POL}\rangle$	$|Y_{HU}^{LogP,H}\rangle$	${|Y_{EU}^{LogP,POL}\rangle }_{A}$	${|Y_{EU}^{POL,H}\rangle }_{A}$	${|Y_{PU}^{POL,H}\rangle }_{B}$
Molecule						
***NG1***	6.0865	5.918	5.308	5.387	5.351	7.210
***NG2***	5.581	5.839	5.399	5.448	4.816	4.578
***NG3***	6.785	6.132	7.526	5.686	1.423	7.234
***NG4***	7.115	6.642	6.037	6.289	5.480	7.765
***NG5***	6.495	7.382	6.853	7.277	6.033	7.629
***NG6***	6.163	7.291	6.426	7.104	7.338	7.647
***NG7***	5.790	7.388	6.087	7.615	6.879	6.547
***NG8***	5.761	7.560	6.330	7.640	7.895	7.447
***NG9***	5.467	5.755	4.786	5.177	7.586	7.303
***R-Pearson***	***0.778***	***0.468***	***0.454***	***0.431***	***0.057***	***0.451***

## Appendix

### A1. More on Catastrophe Theory Background

The foreground of the Catastrophe Theory lies on expressing the Taylor series associated to a smooth function *η*(*c*, *x*), (*c*, *x*) = (*c*_1_,...,*c**_k_*,*x*_1_,..., *x**_m_*), say in its origin (*c*, *x*)= 0 under the form

(A.1) $$\eta (c,x)=j^{s}\eta (c,x)+tayl$$

viewed as the summation of the so called *s-jet* or *s-current*

(A.2) $$j^{s}f(c,x)=\sum_{r=0}^{s}\frac{1}{r!}D^{r}\eta {|}_{0}x^{r}$$

and of its tail generically called here as “*tayl*”. However, in modeling the natural phenomena, unlike the regular (like planets orbits) or continuous ones (with small perturbations included) where the truncation to the *s*-*jet* works fine, many of registered events display sudden (or “catastrophic”) characters, like earthquakes, population growth, or cancer spreading, thus highly requiring for counting of the Taylor tail as well; such need was elegantly resumed by C. E. Zeeman, one of the pioneers of Catastrophe Theory [58], by “allowing the tail of the Taylor series to wag the dog”. When the *tayl* part is becoming important it shapes as the quadratic type dependency on the control × behavior joint space where the original function was defined:

(A.3) $$tayl\propto g^{2}(c,x)$$

This is due to the celebrated Morse’s bifurcation lemma [59] around the so called critical points of the original function, see Equation (3) of the main text, where it actually equivalents the original function with the family of function

(A.4) $$\eta (c,x)\to \tilde{\eta }(g_{1}(c,x),\ldots ,g_{s}(c,x))\pm g_{s+1}^{2}\pm \ldots \pm g_{m}^{2}$$

Here *s*-stays also the co-rank of the Hessian of η (*c*, *x*) in the point (*c*, *x*) = 0. The main question that arises hereby is to try to identify the so called local types of function in a *k*-parametric (control space) family of functions, or, even more, being given a function to identify in its neighborhood the family it belongs to. The solution to this problem was furnished by Thom [1] and then by Arnold [60], by using the powerful concepts of co-dimension and structural tranversality, such that the resulted classification theorem formulates the seven elementary so called catastrophe function of Table 2 as governing all the natural phenomena where the co-dimension is no greater than 4 (four). To better understand that this is indeed covering quite general plethora of natural dynamic systems (with complicated local/turning/singular points modeling sudden changes), enough recalling the heuristic example of the co-dimension for England-Scotland frontier, for instance, that is always equal to 1 (one) no mater one represents the frontier as a line (the road along it), as bidimensional (the road through it on the Earth), as tridimensional (the road through it by plain), or as 4-D (in relativistic vision when the space-time cone is considered as well along it) [61]. It is this co-dimension that controls so powerfully the reduction of all possible power expansions of smooth functions to those seven presented on Table 2; there, one sees the co-dimension number is always equal with the number of parameters from the control space appearing in the Thom polynomials; they, in fact, represent families of functions, *i.e.*, controlling large classes of functions that drive open systems in similar (local) ways. In taxonomical (or algebraically) terms, it is said that although not all functions are typical (or elementary) their families are typical as families; In analytically terms, as all minima through origin look the same (there are said to be typical, and typical like the Morse minima of generalized parabola *x*_1_^2^ + ... + *x**_m_*^2^, eventually after re-parameterization) likewise any transverse path through any non-Morse function that can be found within a family of finite functions looks the same as all other transverse paths in the family (those of Table 2). Even more, the co-rank of those functions (as the co-rank of their Hessian on the critical/singular/turning points) fixes also the minimum of variables that function can be reduced to; for example, if a function of 2011 variables has a critical point of co-rank equal 1, the actual function to be studied is of only 1 variable! This makes the Catastrophe Theory extremely interesting for being applied on QSAR studies, where the available structural variables are listed on hundred pages [62], while in fact one searches for modeling functions that enter natural classes or family of functions with an universal character—as the Thom polynomials are—and therefore aiming to work with appropriate functions with considerable lower number of variables/structural descriptors, see Table 3.

### A2. Catastrophe Theory Implication on Pearson Correlation

Since the transformation of the original smooth function into catastrophe one involves the Morse parabolic polynomials contribution, see Equation (A.4), one may employ this recipe to consider the ordinary QSAR predicted activity, say *Y**^QSAR^*, and of its transformation into the Catastrophe-QSAR one, say *Y**^Γ^*^/^*^QSAR^*, through the Gaussian mapping

(A.5) $$Y_{i=\bar{1,N}}^{\Gamma /QSAR}(X_{1},\ldots X_{M})=Y_{i=\bar{1,N}}^{QSAR}(X_{1},\ldots ,X_{M})\pm Y_{i=\bar{1,N}}^{M/\Gamma /QSAR}(X_{2},\ldots X_{M})$$

with

(A.6) $$Y_{i=\bar{1,N}}^{M/\Gamma /QSAR}(X_{2},\ldots X_{M})=\sum_{j=2}^{M}\frac{1}{\sqrt{\pi }}\text{exp}\left(-\frac{X_{j}^{2}}{4\sigma_{i=\bar{1,N}}}\right)$$

while referring to the running-indices assumed in Table 1. The form (A.5) with (A.6) recovers the original QSAR predicted function/value when all dispersions over all structural variables vanish

(A.7) $$Y_{i=\bar{1,N}}^{\Gamma /QSAR}(X_{1},\ldots X_{M})\overset{\sigma_{i=\bar{1,N}}\to 0}{\to }Y_{i=\bar{1,N}}^{QSAR}(X_{1},\ldots X_{M})$$

thus motivating the actual generalization for treating the natural non-zero dispersive phenomena. On the other side, for higher dispersive values of structural variables (*i.e.*, when their domains of applicability eventually overlap and promote interactions, *i.e.*, the appearance of cross products in Tables 2 and 3) it produces the second order development

(A.8) $$Y_{i=\bar{1,N}}^{\Gamma /QSAR}(X_{1},\ldots X_{M})\overset{\sigma_{i=\bar{1,N}}\to \infty }{\to }Y_{i=\bar{1,N}}^{QSAR}(X_{1},\ldots X_{M})\pm {\tilde{X}}_{2}^{2}\pm \ldots \pm {\tilde{X}}_{M}^{2}$$

under appropriate transformations ${\tilde{X}}_{j=\bar{2,M}}={\tilde{X}}_{j=\bar{2,M}}\left(X_{j=\bar{1,M}}\right)$. However, one can see that in the Catastrophe Theory’s language the first function of the right hand side in (A.8) stays for the 1-jet for the function *Y**^QSAR^*, while the hole expression (A.8) having the Hessian co-rank of order 2 is in full consistence with the maximum co-rank universal unfolding for the polynomials of Table 2.

Next, one likes to check for the effect the Gaussian development (aka the catastrophe transformation) of (A.5) has on the statistical (Pearson) statistical coefficient respecting the QSAR value

(A.9) $$R_{0}=\sqrt{1-\frac{1}{\sigma_{A}}\sum_{i=1}^{N}{\left(A_{i}-Y_{i}^{QSAR}\right)}^{2}},\;\sigma_{A}=\sum_{i=1}^{N}{\left(A_{i}-\bar{A}\right)}^{2}$$

For the sake of clarity we will chose only one sign on (A.5), while the result will not depend on it, and successively obtain

(A.10) $$\begin{array}{c} R_{\Gamma }=\sqrt{1-\frac{1}{\sigma_{A}}\sum_{i=1}^{N}{\left(A_{i}-Y_{i}^{\Gamma /QSAR}\right)}^{2}}\\ =\sqrt{R_{0}-\frac{1}{\sigma_{A}}\sum_{i=1}^{N}{\left(Y_{i}^{M/\Gamma /QSAR}\right)}^{2}+\frac{2}{\sigma_{A}}\sum_{i=1}^{N}\left(A_{i}-Y_{i}^{QSAR}\right)\left(Y_{i}^{M/\Gamma /QSAR}\right)}\\ \leq \sqrt{R_{0}-\frac{1}{\sigma_{A}}\sum_{i=1}^{N}{\left(Y_{i}^{M/\Gamma /QSAR}\right)}^{2}+2\sqrt{\frac{1}{\sigma_{A}}\sum_{i=1}^{N}{\left(A_{i}-Y_{i}^{QSAR}\right)}^{2}}\sqrt{\frac{1}{\sigma_{A}}\sum_{i=1}^{N}{\left(Y_{i}^{M/\Gamma /QSAR}\right)}^{2}}} \end{array}$$

where in the last relation the Cauchy-Schwarz inequality was used:

(A.11) $$\sum_{i=1}^{n}u_{i}v_{i}\leq \sqrt{\sum_{i=1}^{n}u_{i}^{2}}\sqrt{\sum_{i=1}^{n}v_{i}^{2}}$$

Next, in order to draw results that do not depend either on *M*-the number of structure variables nor on *N*-the number of chemicals/molecules involved in a custom QSAR study, one assumes dealing with the same dispersion of the observed activity as well as for each descriptor (the so called homogeneous assumption, σ = σ*_A_* = σ*_i_*, $\forall i=\bar{1,N}$ likely to be valid when dealing with great number of structural descriptors; this way, one actually performs the asymptotic limit *M* → ∞ on (A.6) for all $i=\bar{1,N}$ and recognizes the Poison integral result

(A.12) $$Y^{\infty /\Gamma /QSAR}=\int_{0}^{\infty }\frac{1}{\sqrt{\pi }}\text{exp}\left(-\frac{X^{2}}{4\sigma }\right)dX=\sqrt{\sigma }$$

Accordingly, the inequality (A.10) now reads

(A.13) $$R_{\infty /\Gamma }^{2}\leq R_{0}^{2}-N+2\sqrt{N\left(1-R_{0}^{2}\right)}$$

It may be rearranged upon the second order equation in *N*-chemicals’ space

(A.14) $$-N+2\sqrt{\left(1-R_{0}^{2}\right)}\sqrt{N}+\left(R_{0}^{2}-R_{\infty /\Gamma }^{2}\right)\geq 0$$

whose universal fulfillment leads with the condition

(A.15) $$R_{\infty /\Gamma }\geq 1$$

Since the result (A.15) was obtained within asymptotic conditions regarding the number of structural descriptors and homogeneous dispersion against recorded activity, it can be naturally asserted to its minimum as

(A.16) $$R_{M/\Gamma }\to 1\geq \forall R_{0}$$

thus heuristically proving the superiority for the catastrophe-QSAR modeling over the fashioned QSAR, therefore further motivating the present approach. As numerical illustration of the general prescription of inequality (A.16) the present application confirms it by all one-to-one (*i.e.*, catastrophe-QSAR *vs.* simple QSAR) results reported in Tables 5 and 6.
